# Supplementary material for: Lifestyle Modification: Evaluation of the Effects of Physical Activity and Low-Glycemic-Index Mediterranean Diet on Fibrosis Score
Source: Nutrients. 2023 Aug 10;15(16):3520. doi: 10.3390/nu15163520 (PMC10459797; doi:10.3390/nu15163520)
Supplement: Supplementary file 1 [file nutrients-15-03520-s001.zip › nutrients-2538358-supplementary.pdf]

p r o j e c t

Costo-Beneficio

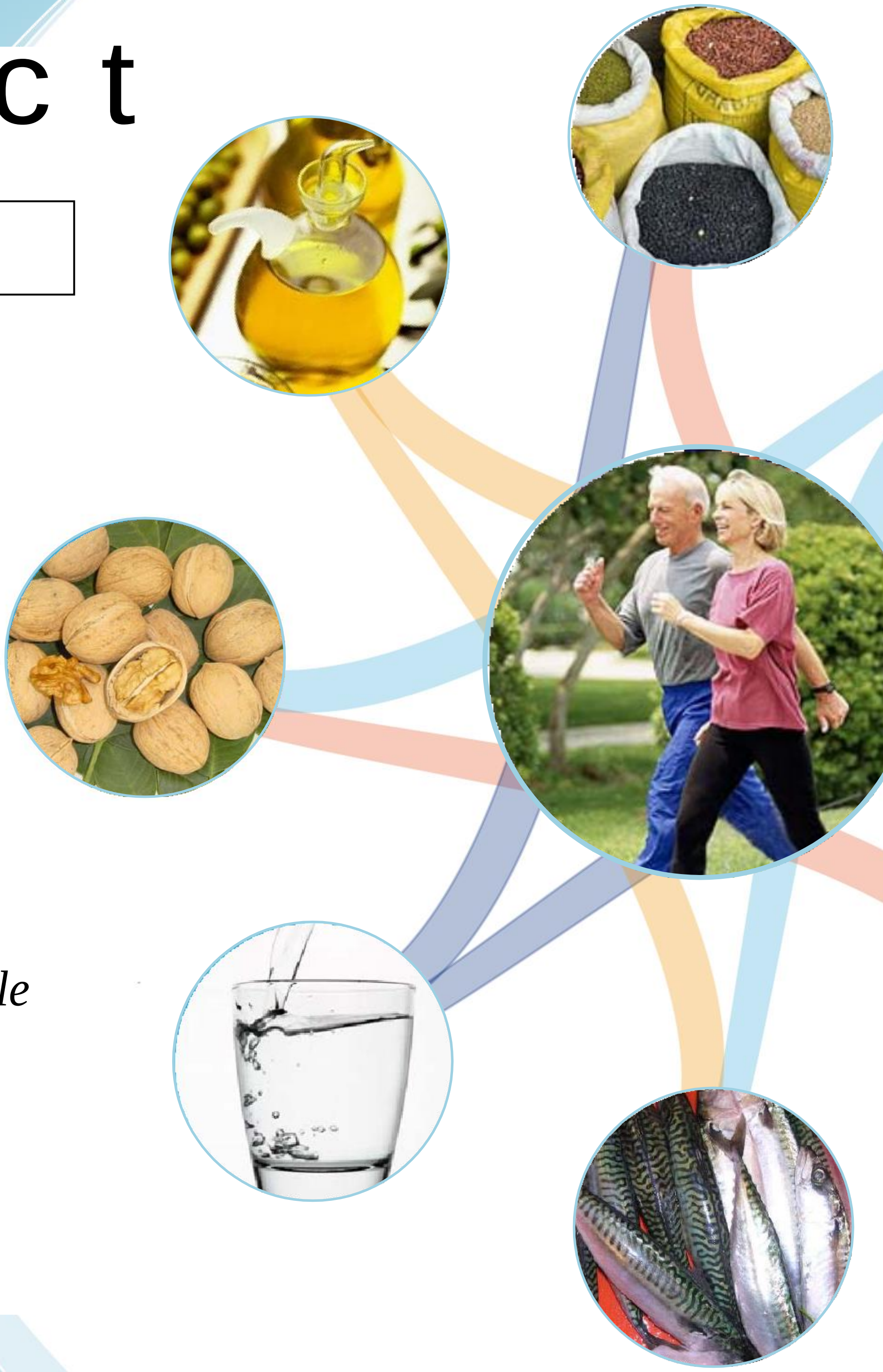

Eat healthy

Adopt a correct lifestyle

Introduction

Your health is in your hands; you build it each day when you go to the supermarket and choose one food rather than another one, whenyou cook a lunch or a dinner. In these moments you do dietary choices which influence your health. A wrong diet can determine the appearance of risk factors for cardiovascular diseases and cancer. One of the steps of these diseases is the Non-alcoholic Fatty Liver Disease (NAFLD) (fat liver).
The Laboratory of Epidemiology and Biostatistics of the IRCCS Saverio de Bellis from Castellana Grotte (Bari) has started up the research project “Analisi Costo-Beneficio di un intervento basato sulla Dieta Mediteranea e l’attività fisica in pazienti diabetic e steatosici” to evaluate the efficacy of the diet and physical activity.

So, we invite you to participate in this project which will help us to understand the efficacy of the diet. Dietary advice described in the following pages are those of Low Glycemic Index Mediterranean Diet and we ask you to follow them. Furthermore, we ask you to record your daily diet in this dietary record as long as the duration of the study. This will permit us to measure adherence levelto the diet we gave you.

WATCH THE SEMAPHORE!

CHECK YOUR DIETARY CHOICES

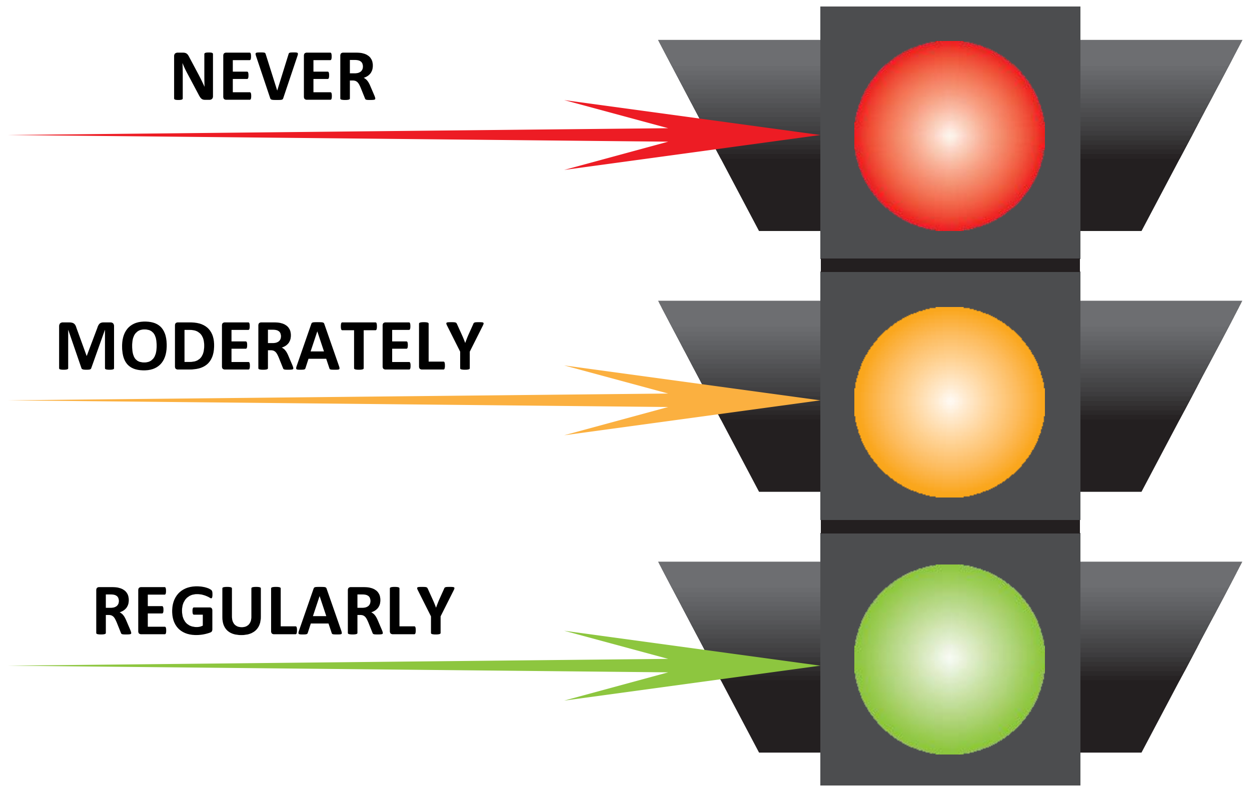

Foods choice and the frequency of their consumption is fundamental to set a correct dietary programm, which day after day should be followed by everyone to keep a satisfying state of health. To direct foods choice towards those which best represent the **LOW GLYCAEMIC INDEX MEDITERRANEAN DIET**, we marked with **GREEN** colour the foods to consume regularly, with **YELLOW** those to consume moderately and with **RED** those to avoid completely.

REGULARLY

- 1- Raw vegetables (lettuce, tomatoes, cucumbers, celery, carrots, radishes, etc.)
- 2- Steamed or boiled vegetables (beets, turnips, chicory, cauliflower, broccoli, etc.)
- 45- Dried pulses without something else (lentils, chickpeas, beans, broad beans, soybeans)
- 46- Own fresh vegetables without something else (peas, beans, green beans)
- 12- Whole wheat pasta with pulses
- 13- Whole wheat pasta with vegetables
- 18- Brown rice with pulses
- 19- Brown rice with vegetables
- 42- Fish (anchovies, sardines, mackerel, etc.)
- 40- Molluscs and crustaceans
- 43- Cod, swordfish, fresh tuna
- 66- Bass bream salmon
- 44- Canned tuna
- 28- Extra virgin olive oil (raw)
- 5- Unsweetened fresh fruit (apples, pears, oranges, grapefruit, kiwi, peaches, etc.)
- 8- Unsweetened nuts (walnuts, almonds)
- 65- Coffee (without sugar or with artificial sweetener)
- 52 -Natural water

MODERATELY

MODERATELY	max
30- Milk and yogurt	1 time/daily
56- Whole wheat biscuits	1 time/daily (3 biscuits)
31- Cheese (parmigiano, cheese, etc.)	2 times/weekly
32- Dairy products (cheese, smoked cheese, cottage cheese)	2 times/weekly
33- Eggs	2 time/weekly (1 egg)
34- White meat (chicken, turkey, rabbit)	2 times/weekly
25- Potatoes (boiled only)	1 time/weekly
22- Whole wheat bread	2 times/daily (a slice)
15- Whole wheat pasta with simple sauce	2 times/weekly (max 80 gr)
4- Sugary fruit (bananas, persimmons, grapes)	1 time/daily (max 1 piece)
62- Dense pure honey	1 time/daily (a teaspoon)
49-wine	2 times/daily (a glass

NEVER

- 11- Not whole wheat pasta
- 17- Not whole wheat rice
- 21- White bread
- 53- Crackers, pretzels, bread sticks, bread and breakfast cereals
- 47- Pizza
- 35- Red meat (beef, veal, pork, horse, etc.)
- 36- Canned meat
- 37- Stew
- 38- Sausages (raw or cooked ham, dried beef, bacon, salami, mortadella, etc.)
- 41- Farmed fish
- 26- French fries or baked
- 67- Fry
- 29- Butter, margarine, mayonnaise, cream
- 9- Fruit in syrup and candied fruit
- 7- Sweet dried fruit (dried figs, dried dates, prunes, raisins)
- 63- Sugar
- 60- Candies and chocolates
- 61- Jams
- 57- Cakes, sweets, pastries
- 58- Croissants, pastries and snacks
- 54- Dry biscuits, toasts
- 55- Biscuits, shortbread
- 59- Ice-cream and water ice
- 51- Alcoholic beverages and spirits (brandy, grappa, liqueurs, whiskey)
- 50- Carbonated/Non carbonated soft drinks (orange juice, coca-cola, fruit juice, etc.)
- 48- Beer

Your Dietary Record

Instructions

- It's fundamental to complete the Dietary Record each day immediately after the meal or as soon as possible.
- The Dietry Record is composed of a series of pages, one for each week. In each weekly page, days from Monday to Sunday are divided in breakfast, lunch, dinner and snack.
- Record all the foods you eat and what you drink during the day (Sunday and holydays included).
- Registration consists in taking note, in correspondence to the meal, the identification number of the food/drink or of the food group consumed belonging to the list we enclosed. If the food you ate isn't included in the list, just write its name in the place reserved to the corresponding meal.

Example:

Here below it is shown an hypothetical day of Mr Rossi's Dietary Record.

After each meal Mr Rossi registered what he ate and drank, so he wrote in the box of the specific meal the correponding number of the food he consumed (by checking the foods list).

At breakfast he drank a cup of milk and coffee without sugar and he ate an apple.

	BREAKFAST	Snack	LUNCH	Snack	DINNER	Snack	PHYSICAL ACTIVITY
MONDAY	30+65<br>5						☐ None Walk<br>☐ Housework<br>☐ Training<br>☐ Other (specify what)<br>☐ ____

At lunch he had a plate of lentils (without pasta) seasoned with raw extra-virgin olive oil, a cod, a plate of raw unseasoned vegetables and he drank two glasses of wine and a glass of natural water.

	BREAKFAST	Snack	LUNCH	Snack	DINNER	Snack	PHYSICAL ACTIVITY
MONDAY			45 + 28<br>43, 1<br>49 + 49<br>52				☐ None Walk<br>☐ Housework<br>☐ Training<br>☐ Other (specify what)<br>☐ ________

In the afternoon he had an apple and drank a coffee without sugar.

	BREAKFAST	Snack	LUNCH	Snack	DINNER	Snack	PHYSICAL ACTIVITY
MONDAY				5<br>65			☐ None Walk<br>☐ Housework<br>☐ Training<br>☐ Other (specify what)<br>☐ ________

At dinner he had a plate of minestrone soup with pasta and seasoned with raw extra-virgin olive oil, a slice of whole bread, a pear and drank two glasses of natural water.

	BREAKFAST	Snack	LUNCH	Snack	DINNER	Snack	PHYSICAL ACTIVITY
MONDAY	30 + 65<br>5		45 + 28<br>43, 1<br>49 + 49<br>52	5<br>65	Minestrone soup<br>+ 28<br>22<br>52 + 52		☐ None<br>☒ Walk<br>☐ Housework<br>☐ Training<br>☐ Other (specify what)<br>________

In the last column, referred to the physical activity carried out during the day, he put a cross in correspondence to the entry "walk".

SURNAME ____________________ LAST NAME ____________________ CODE ____________

MONTH ____________________ WEEK from ____________________ to ____________

	BREAKFAST	Snack	LUNCH	Snack	DINNER	Snack	PHYSICAL ACTIVITY
Monday							☐ None ☐ Walk ☐ Housework ☐ Training ☐ Other (specify what) ____
Tuesday							☐ None ☐ Walk ☐ Housework ☐ Training ☐ Other (specify what) ____
Wednesday							☐ None ☐ Walk ☐ Housework ☐ Training ☐ Other (specify what) ____
Thursday							☐ None ☐ Walk ☐ Housework ☐ Training ☐ Other (specify what) ____
Friday							☐ None ☐ Walk ☐ Housework ☐ Training ☐ Other (specify what) ____
Saturday							☐ None ☐ Walk ☐ Housework ☐ Training ☐ Other (specify what) ____

Sunday							None Walk<br>Housework<br>Training<br>Other (specify what)__________

Ministry of Health

ISTITUTO NAZIONALE di GASTROENTEROLOGIA
DE BELLIS
CASTELLANA GROTTE - ITALIA
ISTITUTO di RICOVERO e CURA a CARATTERE SCIENTIFICO

Research Laboratories
I.R.C.C.S. “Saverio De Bellis”
Castellana Grotte (BA)
tel. + 39 - 080 4994650 / 65
